# Effect of Tourniquet Release Timing on Blood Loss, Surgical Time, and Wound Outcomes in Total Knee Arthroplasty: A Prospective Comparative Study

**DOI:** 10.7759/cureus.89922

**Published:** 2025-08-12

**Authors:** Ali M Hamad, Yazan S Hammad, Ahmad B Maswadeh, Ali Sweidat, Mohammed S Alisi, Jihad Ajlouni

**Affiliations:** 1 Trauma and Orthopaedics, Basildon University Hospital, Basildon, GBR; 2 Sarcoma and Complex Arthroplasty, Royal National Orthopaedic Hospital, London, GBR; 3 Trauma and Orthopaedics, West Middlesex University Hospital, London, GBR; 4 Urology, Hamad General Hospital, Doha, QAT; 5 Orthopaedics, Islamic University of Gaza, Gaza, PSE; 6 Orthopaedics, Jordan University Hospital, Amman, JOR

**Keywords:** blood loss, operative time, total knee arthroplasty (tka), tourniquet release time, wound healing

## Abstract

Background

The timing of tourniquet release in total knee arthroplasty (TKA) remains a topic of debate, with studies suggesting varying impacts on blood loss, operative time, and wound healing. Despite widespread global research, data from our region are limited, and objective assessment tools remain underutilised.

Methods

We conducted a prospective comparative study involving 113 patients undergoing primary TKA. Patients were assigned to one of two groups: Group A (tourniquet released before wound closure) and Group B (released after closure). All surgeries were standardised in terms of technique, prosthesis, and personnel. Outcomes included post-operative haemoglobin drop, intra-articular haematoma volume (measured via ultrasound), operative time, and wound healing at one-week follow-up.

Results

There was no significant difference between the two groups in haemoglobin drop (p = 0.877), intra-articular haematoma volume (p = 0.794), or operative time (p = 0.051). No wound complications were observed in either group.

Conclusion

The timing of tourniquet release, before or after wound closure, did not significantly affect post-operative blood loss, haematoma formation, operative time, or short-term wound healing. Surgeons may safely individualise release timing based on preference and intraoperative factors, provided tourniquet pressure and duration remain within safe thresholds.

## Introduction

Total knee arthroplasty (TKA) is one of the most commonly performed orthopaedic procedures due to its cost-effectiveness and the broad range of conditions it addresses. It is used to treat a variety of degenerative and rheumatologic diseases, as well as certain types of fractures [1-4]. The use of a pneumatic tourniquet during TKA, though widely practised, remains controversial. The ongoing debate centres on whether tourniquets should be used at all, and if so, the optimal timing and duration of application. Despite extensive research, the evidence remains conflicting, making its use largely dependent on surgeon preference.

Tourniquet use offers several advantages, including improved visualisation of the surgical field, reduced intra-operative blood loss, avoidance of technical difficulties, better cement fixation, and shorter operative time [5-7]. Nonetheless, concerns remain regarding its safety, as evidence points to potential adverse outcomes. A meta-analysis and systematic review of 14 randomised controlled trials (RCTs) found higher infection rates, longer hospital stays, greater haemoglobin drop, and increased transfusion requirements in the tourniquet group compared to the non-tourniquet group [8]. Additional studies have reported slower joint functional recovery and a higher incidence of deep vein thrombosis and minor wound complications with tourniquet use [9].

When it comes to the timing of tourniquet release, whether before or after wound closure, literature suggests that early release (before closure) is associated with greater total blood loss, a larger haemoglobin drop, and longer operative time. However, it also correlates with fewer wound complications, lower re-operation rates, and an overall reduction in both minor and major post-operative complications [10-13].

In our study, we compare outcomes based on the timing of tourniquet release, before versus after wound closure. The primary aim was to objectively compare blood loss between early and late tourniquet release in total knee arthroplasty. We hypothesised that early release would result in greater measurable blood loss due to the loss of the tamponade effect. Primary outcomes were assessed using post-operative haemoglobin drop and ultrasound-derived haematoma volume. The former is an established, objective marker of blood loss, while the latter offers a novel, anatomical estimate of internal joint bleeding, enhancing the accuracy of blood loss assessment. Secondary outcomes included operative time and wound healing, selected based on their clinical relevance and frequent association with tourniquet-related complications in prior literature. This study is distinctive in its focus on objectivity, with a standardised surgical team, approach, and prosthesis used throughout. Notably, we employed ultrasound to measure intra-articular haematoma, an evaluation method not previously applied to this topic, to the best of our knowledge.

## Materials and methods

Study design

This prospective, parallel-group, comparative study evaluated the effect of tourniquet release timing on postoperative outcomes in total knee arthroplasty (TKA). It was conducted at a single-centre, tertiary-care teaching hospital (Jordan University Hospital, Amman, Jordan) with Institutional Review Board approval. Participants were recruited and followed up between April 2020 and February 2021. A total of 113 adults scheduled for elective primary TKA were randomly assigned (1:1 allocation ratio) to Group A (n=58), in which the tourniquet was released before skin closure, or Group B (n=55), in which it was released after skin closure. The sample size reflected all eligible patients during the study period; no formal sample size calculation was conducted. No interim analyses or stopping guidelines were planned or conducted. No changes were made to the pre-specified outcomes or analysis plan, and all outcomes reported were defined prior to data collection. Patients or members of the public were not involved in the study design, conduct, or reporting. Due to ethical and institutional constraints, the individual-level dataset and statistical code will not be shared. 

Randomisation and blinding

All patients provided written informed consent prior to participation. Simple randomisation was performed, and surgeons were informed of allocation prior to surgery. Wound assessments were performed by senior registrars blinded to group allocation, and ultrasound evaluations by a radiologist were also blinded. Data analysts were not blinded.

Eligibility criteria

Adults capable of providing informed consent and scheduled for elective primary TKA were eligible. Exclusion criteria comprised rheumatoid arthritis, post-traumatic or revision TKA, and use of anticoagulants or antiplatelet agents (except acetylsalicylic acid).

Surgical technique

All procedures were performed by the same senior surgeon under spinal anaesthesia via a medial parapatellar approach using the Zimmer Biomet NexGen LPS-Flex Knee (Gurgaon, India) posterior-stabilised prosthesis. The operating theatre setup, scrub nurse, and equipment were standardised. Most femoral components were size E, and tibial components were size 4. Tourniquet pressure was set at 120 mmHg above systolic pressure. The femoral canal was sealed with autologous cancellous bone from the distal femoral cut, haemostasis was achieved using diathermy, and the wound was closed in two absorbable layers with skin staples and interrupted vertical mattress sutures, followed by a soft crepe bandage.

Outcomes, post-operative measures, and wound review

The primary outcome, total blood loss, was assessed indirectly via postoperative haemoglobin (Hb) levels on day two, using the final Hb value (g/dL) as the analysis metric (mean and range). Secondary outcomes included residual intra-articular haematoma volume, wound complications (dehiscence, infection, discharge, overall appearance) at the week one outpatient follow-up, and surgical duration (minutes). Wound complications were considered potential harms and assessed non-systematically during routine follow-up. Ultrasound assessment of intra-articular haematoma was performed on postoperative day three, with patients in a supine position and the knee in full extension. A single radiologist, blinded to group allocation, performed all scans. Effusion depths in the suprapatellar pouch, infrapatellar region, and gutters were summed to estimate haematoma volume. Intra-rater reliability was not formally assessed. Post-operatively, Enoxaparin sodium (4,000 IU anti-Xa) was administered 12 hours after surgery for thromboprophylaxis, and full weight-bearing physiotherapy commenced the following morning. Continuous variables are summarised as means and ranges; categorical outcomes as counts and proportions.

Statistical analysis

All randomised participants were analysed according to the intention-to-treat principle. Continuous outcomes (Hb, haematoma volume, surgical time) were compared using Student’s t-test or the Mann-Whitney U test, as appropriate; categorical outcomes (wound complications) were analysed with Fisher’s exact test. No prespecified subgroup or sensitivity analyses were performed. A two-sided p-value of < 0.05 was considered statistically significant.

Funding and conflicts of interest

This study received no external funding or material support. The authors declare no financial or non-financial conflicts of interest; all study conduct and reporting were independent of any funder.

## Results

**Figure 1:**
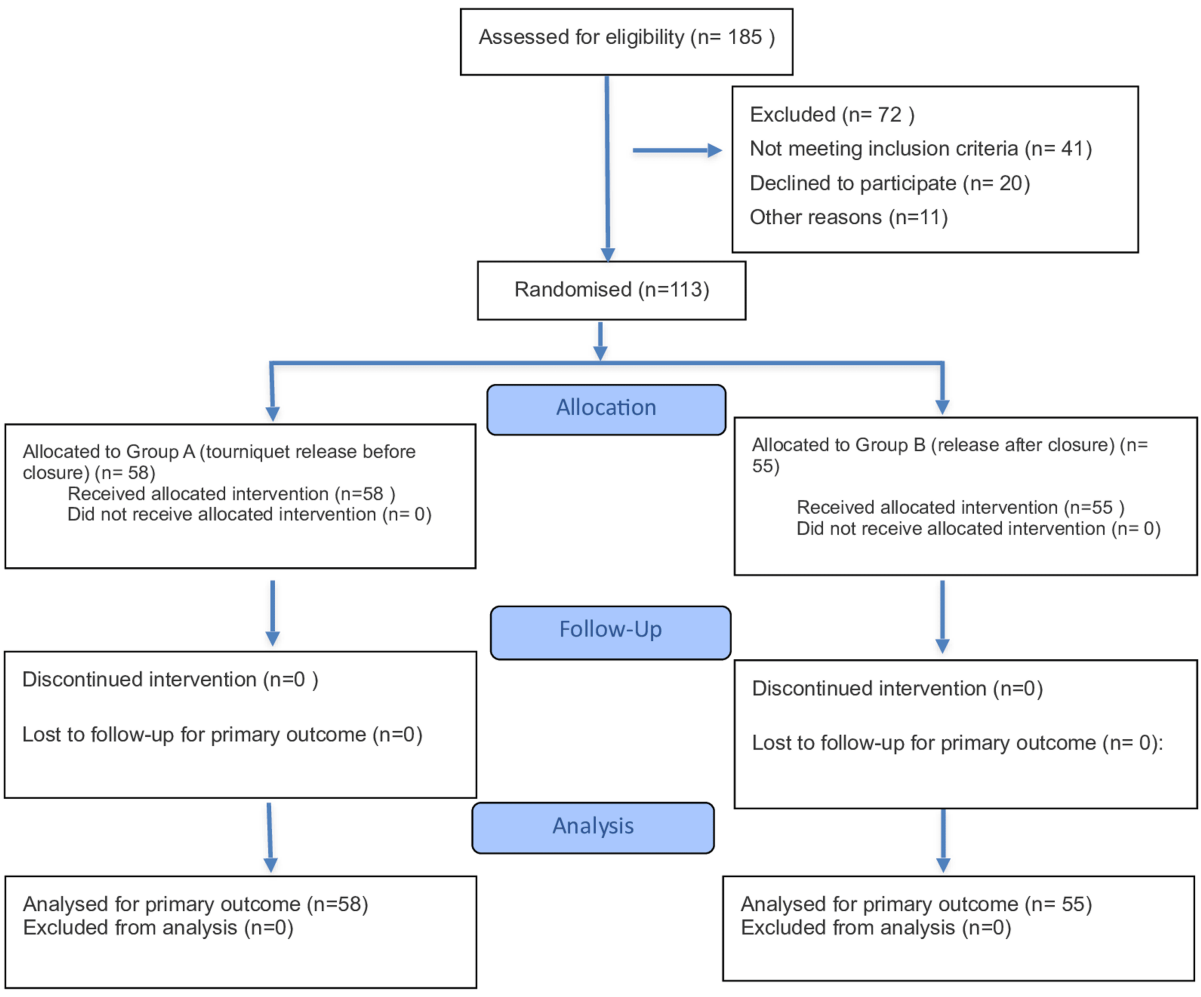
Flow diagram for the study n: number of individuals

Of 185 patients assessed for eligibility, 72 were excluded: 41 did not meet inclusion criteria, 20 declined to participate, and 11 were excluded for other reasons (incomplete workup or scheduling issues). All 113 participants received their allocated intervention and were included in the primary outcome analysis, with a mean age of 66 years. Patients were randomly assigned to two groups: Group A (n = 58), in which the tourniquet was released before wound closure, and Group B (n = 55), in which it was released after closure. No participants were lost to follow-up or excluded after randomisation. All procedures were performed by the same senior surgeon per protocol, with 100% adherence to allocated tourniquet timing. All patients received postoperative thromboprophylaxis with enoxaparin and commenced full-weight-bearing physiotherapy as per protocol. Figure 1 illustrates the participant flow from screening through to analysis.

As shown in Table 1, there were no significant differences between the groups in terms of weight, height, or comorbidities such as hypertension and diabetes. Femoral and tibial component sizes were also comparable, with the E femoral component and size 4 tibial component being the most frequently used in both groups.

**Table 1:** Demographic and prosthesis details by tourniquet release timing n: number, %: percentage

Variable	Released Before Closure (n = 58)	Released After Closure (n = 55)
Mean Age (range)	67 (47–82)	66 (45–82)
Mean Weight (range)	82 (49–110)	83 (45–115)
Mean Height (range)	157 (144–176)	158 (145–170)
Gender		
Female, n (%)	51 (87.9%)	50 (90.9%)
Male, n (%)	7 (12.1%)	5 (9.1%)
Hypertension, n (%)	40 (69.0%)	35 (63.6%)
Diabetes Mellitus, n (%)	10 (17.2%)	16 (29.1%)
Femoral Component Size		
D, n (%)	15 (25.9%)	14 (25.5%)
E, n (%)	27 (46.6%)	32 (58.2%)
Tibial Component Size		
4, n (%)	20 (34.5%)	22 (40.0%)
5, n (%)	17 (29.3%)	17 (30.9%)

Table 2 shows no significant difference in mean surgery duration between groups (p = 0.051). However, tourniquet time was longer in Group B (84 minutes) compared to Group A (63 minutes) (p < 0.01). The mean preoperative haemoglobin was 12.8 mg/dL in Group A and 12.9 mg/dL in Group B (p = 0.579). The mean haemoglobin drop was -2.36 mg/dL in Group A and -2.31 mg/dL in Group B (p = 0.877), showing no significant difference.

**Table 2:** Intra-operative and post-operative outcomes by tourniquet release timing Values are presented as mean with range between parentheses. Haemoglobin is measured in mg/dL. Intra-articular haematoma volume measured by ultrasound on postoperative day three in millilitres in full extension. p-values calculated using independent t-tests.

Variable	Released Before Closure (n = 58)	Released After Closure (n = 55)	p-value
Surgery time (minutes; range)	78 (55–100)	83 (55–110)	0.051
Tourniquet time, (minutes; range)	63 (45–90)	84 (45–110)	<0.01
Preoperative haemoglobin (mg/dL; range)	12.8 (9.8–16.6)	12.9 (10.2–17.6)	0.579
Haemoglobin drop (mg/dL; range)	-2.36 (-0.6 to -4.85)	-2.31 (-0.5 to -4.20)	0.877
Intra-articular haematoma by ultrasound at POD 3 (ml; range)	97 (0–400)	95 (6–334)	0.794

Regarding intra-articular haematoma formation, assessed by ultrasound on postoperative day three, the mean volume was 97 mL in Group A and 95 mL in Group B (p = 0.794). While mean volumes were similar, Group A had a wider range (0-400 mL) compared to Group B (6-334 mL).

At the one-week post-operative follow-up, no cases of wound dehiscence, infection, or delayed healing were observed in either group. No adverse events, including deep vein thrombosis (DVT), pulmonary embolism (PE), or systemic complications, were reported in either group during the study period.

## Discussion

The use of a tourniquet in total knee arthroplasty (TKA) has long been a topic of debate, with conflicting evidence surrounding its effects on blood loss and postoperative complications. Although this subject has been widely studied globally, data from our region remains limited. To address this gap, we conducted a prospective comparative study designed to maximise objectivity through strict standardisation of surgical variables and the use of ultrasound to quantify internal blood loss, an assessment method that is unreported in the current literature.

The primary finding of this study was that there was no statistically significant difference in blood loss between the two groups, as measured by post-operative haemoglobin drop and intra-articular haematoma volume. This aligns with the findings of a recent study by Ghazni Khan et al., who similarly reported no significant difference in total blood loss when comparing early and late tourniquet release during TKA [14]. A meta-analysis of 16 randomised controlled trials (RCTs) examining blood loss, complications, and other variables also found no statistically significant difference in blood loss between the groups; however, the absolute volume of blood loss was higher in cohorts where the tourniquet was released prior to wound closure [13]. Interestingly, another meta-analysis of 16 RCTs reported no significant difference in measured postoperative blood loss, haemoglobin drop, or haematocrit drop but did find a statistically significant increase in total blood loss and postoperative transfusion rates in the early release group. Total blood loss was calculated as a composite of intraoperative, postoperative, and hidden losses, the latter attributed to factors such as internal tissue bleeding and haemolysis [11]. Multiple other studies have supported the finding that early tourniquet release results in higher total blood loss [15-18].

On the other hand, delayed tourniquet release has been associated with impaired wound healing and an increased rate of both minor and major post-operative complications [11-14,19]. In our study, however, no wound complications were observed in either group at the one-week follow-up. It is also worth noting that both tourniquet pressure and duration of application influence the rate of complications, with evidence suggesting more favourable outcomes when pressure is maintained below 225 mmHg and application time is limited to under 100 minutes [20,21]. This may be attributed to the effect of tourniquets in reducing oxygen supply around the wound, leading to ischaemia-reperfusion injury, which disrupts the microvasculature and impedes normal wound healing [22].

The final parameter we assessed was operative time. Our results showed no significant difference between the two groups. However, some studies have reported longer operative times when the tourniquet is released before wound closure compared to after closure [13,18,22,23]. In a meta-analysis of eight RCTs, Tai et al. found that early release did not reduce operative time compared to no tourniquet use, whereas late release was associated with a shorter operative time [23]. On the other hand, other studies have suggested that not using a tourniquet at all may actually result in shorter operative times compared to using one [24-26].

To further clarify the clinical interpretation of our negative findings, we calculated mean differences with 95% confidence intervals and estimated effect sizes for the primary and secondary outcomes. The difference in haemoglobin drop between groups was -0.05 g/dL (95% CI: -0.42 to 0.32), with a negligible effect size (Cohen’s d = -0.05). For intra-articular haematoma volume, the mean difference was 2 mL (95% CI: -31.65 to 35.65; Cohen’s d = 0.02), also indicating a trivial effect. The operative time difference was -5 minutes (95% CI: -9.65 to -0.35), corresponding to a small-to-moderate effect size (Cohen’s d = -0.40). These results suggest that even if statistically non-significant, the observed differences were unlikely to be clinically meaningful, particularly in the case of blood loss and haematoma formation. Nonetheless, as our study was not powered for equivalence testing, the absence of statistical significance should not be interpreted as proof of equivalence.

Our study presents several limitations. Although no formal sample size calculation was performed, we addressed this by reporting effect sizes, mean differences, and 95% confidence intervals for key outcomes to aid interpretation of clinical relevance. While the sample size was sufficient to detect moderate differences, it may not have captured more subtle effects. The follow-up period was limited to one week and lacked systematic assessment, potentially overlooking delayed wound complications. Additionally, although intra-articular haematoma was assessed using ultrasound, intra-rater reliability was not formally evaluated, and intra-operative blood loss was not separately measured, both of which may have affected the precision of total blood loss estimation. Finally, as a single-centre study, the generalisability of our findings to other surgical teams or populations may be limited.

Despite these limitations, this study provides a unique and objective contribution to the current literature by incorporating ultrasound-based assessment of haematoma volume, an approach rarely used in similar studies. Additional strengths include a standardised surgical protocol with a single surgeon, a fixed prosthesis type, and a consistent surgical team, which minimised inter-operator variability and enhanced internal validity. Blinded assessment of outcomes by both the ultrasound examiner and wound reviewers helped reduce measurement bias. The study also achieved 100% adherence to the intervention protocol, with all participants receiving their assigned allocation. Finally, efforts to analyse participants according to their initial group allocation were made to strengthen methodological rigour and reliability of the findings. These strengths support a more precise evaluation of the impact of tourniquet release timing in total knee arthroplasty.

## Conclusions

This prospective comparative study found no significant difference in postoperative blood loss, intra-articular haematoma formation, wound complications, or operative time between early and late tourniquet release in total knee arthroplasty. While existing literature suggests early release may increase total blood loss and late release may negatively impact wound healing, our results did not demonstrate clinically significant differences between the two approaches. This study indicates that the timing of tourniquet release has minimal influence on short-term outcomes. However, given the absence of long-term outcomes and follow-up, and our sample size, further research is necessary to establish clear clinical guidance on the timing of tourniquet release. 
